# Positron Annihilation Spectroscopy Study of Carbon-Vacancy Interaction in Low-Temperature Bainite

**DOI:** 10.1038/s41598-020-57469-x

**Published:** 2020-01-16

**Authors:** Rosalia Rementeria, Ricardo Domínguez-Reyes, Carlos Capdevila, Carlos Garcia-Mateo, Francisca G. Caballero

**Affiliations:** 1 Department of Physical Metallurgy, Spanish National Center for Metallurgical Research (CENIM-CSIC), Avda. Gregorio del Amo 8, E-28040 Madrid, Spain; 2 Present Address: ArcelorMittal Global R&D, SLab – Steel Labs, Calle Marineros 4, E-33490 Avilés, Asturias Spain; 3 Departamento de Física, Universidad Carlos III de Madrid, Avda. de la Universidad 30, E-28911 Leganés, Madrid Spain

**Keywords:** Structural materials, Theory and computation

## Abstract

Nano-scale investigations of bainitic structures formed at temperatures below 350 °C have shown that the bainitic ferrite lattice is super-saturated in carbon. A high density of intrinsic defects would be playing a part in the carbon-supersaturation levels detected. In this work, the role of C−vacancy complexes on carbon-supersaturation in low temperature bainite is investigated by means of Positron Annihilation Spectroscopy. Results reveal the presence of a significant amount of monovacancies in the structures that plays an important role on the formation of carbon clusters in the ferrite lattice of nano-scale bainitic structures.

## Introduction

Low-temperature bainite, mainly consisting of nano-scale plates of bainitic ferrite and carbon-enriched regions of austenite, is formed by austenite decomposition at temperatures below 350 °C in high-carbon high-silicon steels^1,2^. Diffraction-based techniques^3–6^ together with extensive Atom Probe Tomography (APT) data analyses of these structures^7–10^ revealed a measurable tetragonality and an important amount of carbon in solid solution in the bainitic ferrite which remain even after prolonged heat treatment or subsequent tempering^10^.

Since the quantification of solid solutions in APT datasets is not trivial^11,12^, the APT analyses approach was recently reconsidered^13^ finding that the amount of carbon that remains in solid solution in bainitic ferrite is significantly smaller than that derived from the *c*/*a* ratio of bct bainitic ferrite as determined by diffraction studies. Complementary Transmission Electron Microscopy (TEM)^14^ showed that the increased tetragonality detected by diffraction analysis is the result of carbon clusters considered to be carbide embryos^15^, with a composition close to the stoichiometric *α*″-Fe_16_C_2_, embedded in a carbon-depleted matrix, as in the early stages of ageing of martensites^16,17^.

In the course of the displacive transformation of the austenite, large plastic strains lead to high concentrations of intrinsic defect, with carbon interstitials binding strongly to vacancies^18^. In addition, low temperature bainite formation involves a rapid quenching after austenitization, resulting in a non-equilibrium supersaturation of thermal carbon-vacancy complexes retained in the austenite, due to their increased diffusion barriers^19,20^. It has long been known that C-vacancy complexes can also act as precursors for carbide precipitation during tempering of martensite^21^, although it remains unclear if the presence of C-vacancy bonds affects carbon clustering in unstable structures. In this work, the nature of the defects and their stability in relation with the alloying elements and the transformation temperature is investigated in low temperature bainite by Positron Annihilation Spectroscopy (PAS).

PAS is sensitive to lattice defects^22^ and is also capable of detecting the atomic environment of positron annihilation sites^23^. Open-volume defects, such as vacancies, vacancy-clusters, or solute–vacancy complexes, are effective traps for thermalized positrons in metals. The annihilation radiation of a positron trapped in a defect conveys information about the nature of the defect. The positron lifetime distribution provides information on the type, size and concentration of the defects. In addition, information about the chemical surround of the positron annihilation site can be obtained using the annihilation radiation peak at 511 keV through its Doppler broadening, as it depends on the momentum of the electrons annihilating with the thermalized positrons^24,25^. This assessment is of prime interest to understand carbon super-saturation in bainitic ferrite and its relation to lattice defects.

## Materials and Experimental Procedure

The chemical composition of the studied alloy is Fe-0.66C-1.45Si-1.35Mn-1.02Cr-0.10Ni-0.24Mo in mass fraction. Each sample was austenitized at 900 °C, for 15 min and subsequently cooled at 50 °C/s to the bainite reaction temperature, between 220 and 300 °C, held for enough time to ensure the completion of the transformation, and subsequently quenched to room temperature. Transformation kinetics and structure description have been reported in previous works, see refs. ^10,13,26^ In addition, martensite and pearlite structures were obtained after fully austenitization at 900 °C for 15 min and subsequent continuous cooling at 50 °C/s and 0.1 °C/s, respectively. Bainitic structures are encoded as Bx, where x is the bainite transformation temperature, while the pearlite and martensite structures are encoded as PER and MAR, respectively (See Table 1).

**Table 1:** PLS results for the structures formed in the studied steel.

Sample	Heat treatments	Microstructure	τ (ps)
PER	Slow cooling at 10 °C/s	Pearlite	151 ± 3
M	Fast cooling at 50 °C/s	Martensite	159 ± 3
B220	Isothermal heat treatment at 220 °C for 24 h.	Bainite	164 ± 3
B220 +6d^*^	Isothermal heat treatment at 220 °C for 168 h.	Bainite	162 ± 3
B250	Isothermal heat treatment at 250 °C for 8 h.	Bainite	165 ± 3
B300	Isothermal heat treatment at 300 °C for 5 h.	Bainite	160 ± 3

^*^The suffix ‘+6d’ indicates that the sample was overaged at the transformation temperature during 6 days after completion of bainite transformation.

The PAS experiments were performed in a fast-fast spectrometer configured in coincidence. The time resolution was determined using a ^22^Na source (Kapton sealed) placed between a pair of reference samples, resulting in 230 ps (FWHM). Positron lifetime spectra (PLS) with a total count >10^6^ were fitted subtracting the corrections due to positron annihilation in the ^22^Na source. Reference samples of pure Fe annealed at 800 °C during 9 hours, and Si single crystals were used to determine the instrumental time resolution and the source correction for the lifetime spectra. The source contribution was determined as; 382 ps with 13.6% intensity along with another component of ~1.2 ns, with 0.2% intensity, corresponding to the interaction with the surface of the source. Spectra were fitted with the PATFIT-88 package^27^.

Coincidence Doppler Broadening (CDB) measurements were performed placing the samples at the center of a face-to-face configuration of two high-purity germanium detectors (HPGe) set in timing coincidence. A series of individual spectra, with a count number >10^6^ in a 512 × 512 coincidence matrix each, were used to achieve cumulative spectra (after checking the absence of electronic shift) with 1 × 10^7^ counts in the strip centered on the matrix diagonal (defined as $2{m}_{0}{c}^{2}-1.6\,{\rm{keV}} < {E}_{1}+{E}_{2} < 2{m}_{0}{c}^{2}+1.6\,{\rm{keV}}$, where *E*_1_ and *E*_2_ stand for the energies of the pair of annihilating photons, ${m}_{0}=5.110\times {10}^{2}\,\text{keV}/{c}^{2}$ is the electron rest mass and *c* is the speed of light). The spectra were re-binned from 512 × 512 to 40 × 40 energy groups with a bin width of 2.5 × 10^−3^ *m*_0_*c* to decrease the statistical fluctuations of the data in the high momentum region. Aiming to highlight the differences between the spectra, ratio curves were obtained (after normalizing) dividing by the corresponding CDB spectrum of a pure annealed Fe reference sample.

## Results and Discussion

Vacancies are the dominant self-defects at room temperature in metals. In the case of Fe, theoretical calculations indicate that di-vacancies, tri-vacancies, tetra-vacancies, compact clusters and small voids are stable^28–31^ and form various atomic configurations which are thought to be the nuclei for dislocation loops and new-types of crystal lattices^29^. The common interstitial atoms in steels, i.e., H, C and N, interact with point defects, thereby reducing their diffusivities but also modifying the migration properties of those point defects^30,32–34^. In both low and high carbon steels, vacancies have a carbon dimer bound to them^18,33,35–37^, where carbon does not occupy a vacant Fe lattice site, but a position close to its preferred interstitial site (octahedral interstice) at one of the cube faces bounding the vacancy^35^.

Theoretical models predict that the formation of such vacancy-carbon complexes significantly reduces the vacancy diffusivity in bcc Fe, exhibiting non-Arrhenius behavior^32,33^. Besides, carbon diffusivity decreases with increasing carbon content and becomes negligible when the carbon concentration exceeds twice that of vacancies^34^. The controversy on vacancy migration calls into question whether mobile carbon interstitials are trapped by stable monovacancies, or viceversa. This ‘chicken and egg’ situation is translated into a carbon concentration in Fe above that expected from the thermodynamic equilibrium. Additionally, substitutional solute atoms can act as traps for vacancies and/or modify the structure and therefore the stability and migration properties of point defect clusters^38^.

Table 1 presents the PLS results in the structures obtained from the studied steel. For each of the structures, more than 10^6^ single annihilation events were recorded in order to obtain the complete positron lifetime spectrum *N*(*t*),

1 $$N(t)={\sum }_{i=1}^{k+2}{I}_{i}exp(-\frac{t}{{\tau }_{i}})$$

where the *k* different components contributing to the positron trapping with 2 source corrections components in the spectra with the individual lifetimes *τ*_*i*_, and intensities *I*_*i*_.

Results in Table 1 were obtained from data fitting to Eq. (1) by minimizing the Chi-square function defined as:

2 $${{\rm{{\rm X}}}}^{2}={\sum }_{i}\frac{{({e}_{i}-{f}_{i})}^{2}}{{f}_{i}}$$

where in Eq. (2), *e*_*i*_ are the experimental values and *f*_*i*_ are the values obtained fitting Eq. (1) after convoluting each component with the time resolution function of the spectrometer, 230 ps (FWHM). This function was then normalized based on the number of degrees of freedom and the number of fitting parameters on each case obtaining a result for the normalized Chi-square function better than 6 × 10^−3^. Fitting provided a lifetime precision of 3 ps.

Values in Table 1 for the martensitic (M) and bainitic microstructures formed at different temperatures and times (B220, B220 +6d, B250 and B300) show a consistent lifetime around 160 ps, whereas the pearlitic structure (PER) shows a slightly lower PLS value. The consistency of the PLS values for all bainitic microstructures, even for the over-aged sample, evidences the lack of influence of the bainite reaction temperature or the over-aging process in the nature of the positron traps.

As Supplementary Information illustrates, fitting the data to a two-component model using the bulk lifetime (~110 ps^39^) and single vacancy lifetime (~175 ps^40^), following methodology reported elsewhere^41,42^, was unsuccessful even when fixing the lifetime parameters (with the consideration of a two-state model that would change the value measured for the bulk). This means that the 160 ps lifetime corresponds to a type of defects whose lifetime is shorter than the lifetime associated to Fe single vacancies, and that positron trapping saturates at those defects, excluding the possibility of annihilation at the bulk or at positions related to the deformation caused by interstitials in the bulk. A lifetime shorter than that corresponding to annihilation in Fe single vacancies is associated to the annihilation in a C−vacancy pair formed by the capture of migrating vacancies by carbon atoms^43,44^.

The experimental lifetime obtained for these C-vacancy pairs has been reported to be ~160 ps^45^. The positron trapping rate for the C−vacancy pair and the mono-vacancy in the Fe−C system can be considered as equal^46^. Vehanen *et al*.^43^ stated that considering the same trapping rate for vacancies in Fe-C alloys and in C-vacancy pairs, could possibly lead to a slight underestimation of the pair concentration, suggesting that even when the trapping rates might not be equal, they must be similar. This means that the positron trapping process is analogous in C−vacancy and in Fe−vacancy positions, so the elemental affinity for positrons takes a major role in the process. The fact that carbon has a high affinity for positrons^46^ explains the impossibility of determining the annihilation component in the bulk of the material, rather than having a high concentration of defects that would produce saturation in the positron trapping.

**Figure 1:**
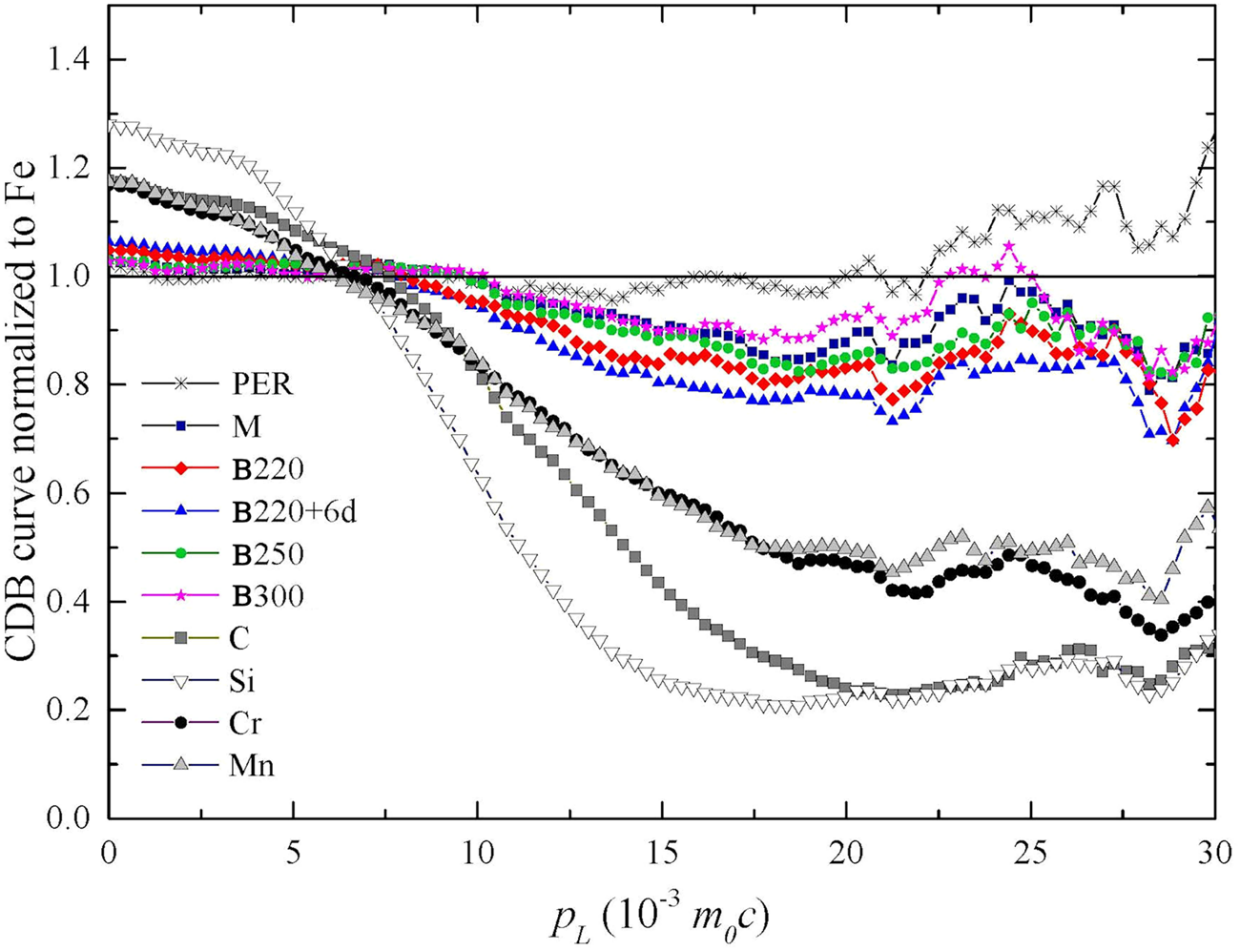
CDB ratio spectra for pearlite (PER), martensite (M) and bainite formed at different temperatures (B220, B250 and B300) obtained after different heat treatments (see Table 1 for details) from the studied steel, normalized by the CDB spectrum of a pure annealed Fe reference sample. The normalized CDB ratio curves for C, Si, Cr and Mn are shown for comparison. The suffix ‘+6d’ indicates that the sample was held at the transformation temperature during 6 days after completion of bainite transformation.

The characteristics of the CDB ratio curves for the microstructures obtained from the studied steel are shown in Fig. 1, along with the ratio curves for the major alloying elements (C, Si, Mn and Cr). All the CDB curves are normalized to the one for a pure annealed Fe reference sample as this is the predominant element in the alloy. CDB curves normalized to the other component of the alloy were also obtained attempting to highlight some behaviors, but the results was substantially less clear than the CDB curves normalized to Fe, probably because of the high number of components of the alloy and their reduced relative amount.

The deviation between different curves of the same sample in same conditions corresponds to the uncertainty of the experiment. Deviations from the unity in the CBD ratio curves are the result of positron trapping in defects with a configurational surrounding that can contain, either concurrently or separately, C, Si, Cr or Mn. Shifting in the CDB ratio spectra of the analyzed structures towards the CDB signature curve of certain pure alloying element (here C, Si, Cr or Mn) can be only interpreted as an association of the defects in the structures with that alloying element. The possibility that the shift of CDB curve could be related to a precipitation process can be ruled out since carbides are formed by para-equilibrium growth (i.e. without partitioning of substitutional elements across carbide particles) during bainite reaction at low temperature^7^. Parameters *W* and *S* associated to the CDB data were calculated in an attempt to improve the understanding of the nature of the positron traps in the alloy; but *W-S* plots provided no conclusive information. This might be caused by the fact that the alloy present positron traps associated to different structures resulting in a complex *W-S* plot that cannot interpreted with the classic *W-S* parameters theory. This could imply that the saturation in the positron trapping evidenced in PLS might be caused by this existence of several kind of structures associated to the traps more than a high amount of a single type of defect.

The CDB curve for the pearlitic microstructure (PER) evidences a low amount of vacancy-type defects, given that it stays close to unity for all momentum regions meaning that almost matches the curve distribution of the annealed “defect-less” Fe. On the contrary, the shape of the CDB curves of the martensitic and bainitic structures is similar while gets away from the unity, implying that the defect distribution has no major chemical change on its surroundings despite the microstructure obtained. The shape of the CDB curves can be considered to have two contributions, C and/or Si, and Cr and/or Mn. The CDB curves are based in the electron configurations, so that elements of the same chemical group share the same signature curve at high-momentum regions (above 20·10^−3^ *m*_0_*c*), as seen in the CDB curves for carbon and silicon in Fig. 1. The only difference is related to the annihilation with electrons coming from the core of the atom that modifies the shape of the curve at mid-momentum (below 20·10^−3^ *m*_0_*c*) and low-momentum region (below 10·10^−3^ *m*_0_*c*). Thus, the contribution of silicon (with external electron configuration 3s^2^3p^2^) and carbon (with external electron configuration 2s^2^2p^2^) to the CDB curves for the studied alloy can be only distinguished by the analysis of the mid and low-momentum region in which carbon presents lower slope and values nearer to the unit as compared to silicon.

Since the microstructures studied present a CDB curve very similar among them, and the slope of these curves at the low-momentum region is analogous to that of carbon, it might be derived that the contribution of silicon is marginal. No evidences of an interaction between silicon and carbon could be concluded form these experimental results, as suggested by previous theoretical calculations^47^.

The Cr and Mn contents of the steel are comparable. These two alloying elements have also similar electron configurations within the ground state, 4s^1^3d^5^ and 4s^2^3d^5^, respectively, and similar positron affinity; resulting in analogous CDB spectra that are almost impossible to distinguish in any momentum region (see Fig. 1). Using additional CDB curves referenced to Cr and Mn to settle this influence was unsuccessful due to the great similarity of both reference curves requiring additional arguments to clarify the role of each component of the alloy. The contribution of these elements is expected to be lower than the carbon and silicon contributions due to the lower content in the alloy. It is known that Mn and C form dipoles in both the fcc and bcc lattices of Fe due to their high binding energy (~0.36 eV)^48–51^, which is increased by the presence of Si^52^. Experimental results in the Fe−C−Mn system indicate that the solubility of carbon in bcc-Fe below 550 °C increases with an increase in the Mn content of the steel^51^ and the presence of Mn−C dipoles has been suggested to be a contributing factor to the observed carbon super-saturation in bainitic ferrite^53^. Therefore, it can be argued that the major contribution to the studied steel CDB curve is more dominated by Mn than by Cr because of the presence of carbon in the environment of vacancies, as evidenced by PLS and CBD.

Overall, the CDB curves of the bainitic structures in Fig. 1 shifts towards the carbon CDB signature curve as the transformation temperature decreases. The largest differences among the bainitic structures are observed in the mid-momentum region and part of the high-momentum region. The largest shift from unity is observed for the bainitic structure overaged at 220 °C for 6 days followed, in descending order, by the bainitic structures formed at 220, 250 and 300 °C. Thus, the present results indicate that a decrease in the transformation temperature results in larger amounts of carbon (with a contribution of Mn) bound to vacancy-type defects. Lower transformation temperatures are translated into higher amounts of bainitic ferrite with also higher levels of carbon super-saturation in bainitic ferrite^9,10^. Therefore, the presence of C-vacancy complexes should contribute to the carbon super-saturation levels detected in bainitic ferrite. It is speculated that the low mobility of the C-vacancy complexes in the bainitic ferrite matrix hinders the carbon decarburization of bainitic ferrite promoting α“-type ordering processes observed in APT reconstructions reported elsewhere^14^.

## Conclusions

Positron Lifetime Spectroscopy measurements have revealed the presence of a significant amount of monovacancies in low temperature bainite, that would assist in retaining carbon in bainitic ferrite by the formation of C−vacancy complexes. The low mobility of these complexes in the ferrite impedes the full partitioning of carbon towards the austenite/ferrite interface promoting carbon clustering phenomena in the ferritic phase formed at low temperature. Complementary simulations studies are required to explain the observed C−vacancy complexes stability. For that purpose, first the development of a new Fe-C interatomic potential to use in Molecular Dynamics and Kinetic Monte-Carlo simulations is required. This investigation is in progress.

## Supplementary information

Supplementary Information
