# Supplementary material for: Positron Annihilation Spectroscopy Study of Carbon-Vacancy Interaction in Low-Temperature Bainite
Source: Sci Rep. 2020 Jan 16;10:487. doi: 10.1038/s41598-020-57469-x (PMC6965194; doi:10.1038/s41598-020-57469-x)
Supplement: Supplementary file 1 — Supplementary Information [file 41598_2020_57469_MOESM1_ESM.docx]

**Supplementary Information**

**Positron Annihilation Spectroscopy Study of Carbon-Vacancy Interaction in Low-Temperature Bainite**

Rosalia Rementeria^1,2^, Ricardo Domínguez-Reyes^3^, Carlos Capdevila^1^, Carlos Garcia-Mateo^1^, and Francisca G. Caballero^1*^

^1^Department of Physical Metallurgy, Spanish National Center for Metallurgical Research (CENIM-CSIC), Avda. Gregorio del Amo 8, E-28040 Madrid, Spain.

^2^ArcelorMittal Global R&D, SLab – Steel Labs, Calle Marineros 4, E-33490 Avilés, Asturias, Spain (current address).

^3^Departamento de Física, Universidad Carlos III de Madrid, Avda. de la Universidad 30, E-28911 Leganés, Madrid, Spain.

*corresponding author: fgc@cenim.csic.es


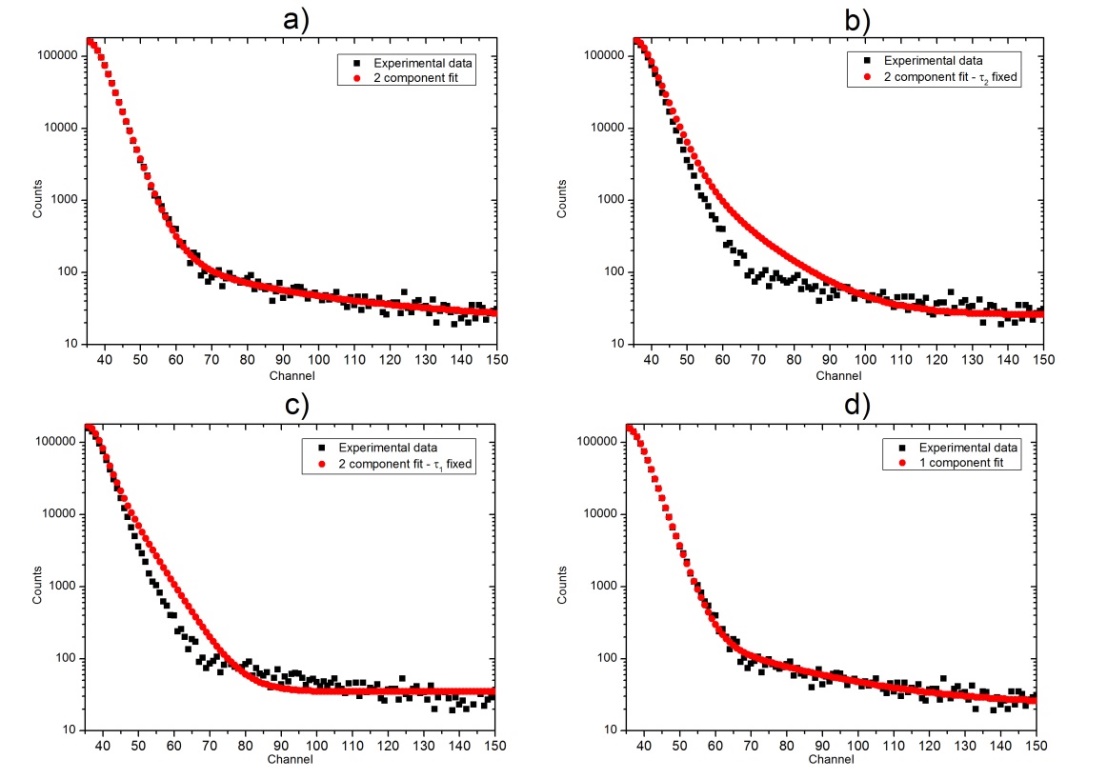


**Figure S1:** Comparison between PLS results for bainitic structure transformed at 300ºC for 5 h (B300 sample) and data fitting considering: a) two free components, b) two components with individual lifetime *τ_2_* fixed at Fe-vacancy lifetime of 175 ps, attempting to reproduce a two-state model, c) two-components with individual lifetime *τ_1_* fixed to Fe bulk lifetime of 110 ps, and d) one component. Details on the data fitting method are reported elsewhere [S1, S2].

**Table S1**: Corresponding lifetime components from PLS fitting with different conditions along with their respective intensities *I_i_* and normalized Χ^2^. The initial values used for data fitting were *τ_1_*=110 ps and *τ_2_*=175 ps for two components fitting, and *τ_1_*=175 ps for one component fitting. Same results were obtained for different set of initial values.

| Type of fit | *τ_1_* (ps) | *I_1_* (%) | *τ_2_* (ps) | *I_2_* (%) | Normalized Χ^2^ |
| --- | --- | --- | --- | --- | --- |
| Two components | 159±3 | 99±1 | 390± 150* | 1±1* | 0.006 |
| Two components - τ_2_ fixed | 9±7 | 4±2 | 175^a^ | 96±2 | 0.472 |
| Two components - τ_1_ fixed | 110^a^ | 96±2 | 897±16 | 4±2 | 0.475 |
| One component | 160±3 | 100^b^ | -- | -- | 0.005 |

*Component with intensity compatible with no error bar with lifetime value associated to the source correction.

^a^ No error as consequence of being a fixed parameter.

^b^ No error as consequence of being the only component of the fit.

References:

[S1] R. Domínguez-Reyes, B. Savoini, M. A. Monge, A. Muñoz and C. Ballesteros, *Adv. Eng. Mat.*, 19, 1527 (2017).

[S2] R. Domínguez-Reyes, M. A. Auger, M. A. Monge and R. Pareja, *Phil. Mag.* 97, 833 (2017).
